# [AU(DIEN)Cl]Cl_2_: Exchange Phenomena Observed by ^1^H and ^13^C NMR Spectroscopy

**DOI:** 10.1155/MBD.1999.261

**Published:** 1999

**Authors:** Sabine L. Best, Zijian Guo, Milos I. Djuran, Peter J. Sadler

**Affiliations:** 1 Department of Chemistry Birkbeck College University of London 29 Gordon Square London WC1H OPP UK; 2 Department of Chemistry University of Edinburgh West Mains Road Edinburgh EH9 3JJ UK

## Abstract

The solution behaviour of the square-planar gold(III) complex [Au(dien)Cl]Cl_2_ (dien = 1,5- diamino-3-azapentane) was investigated by ^13^H and ^13^C NMR spectroscopy. We have found that ^1^H NMR spectra of [Au(dien)Cl] Cl_2_ are characterised by exchange behaviour over the whole pH range, and some exchange effects are also seen in ^13^C NMR spectra of the deprotonated hydroxo derivative of the complex in alkaline solution. An exchange rate of > 378 s^-1^ was determined from ^1^H NMR spectra at pH∗^2^ (coalescence temperature ^40^C°). In slightly acidic solutions of the complex, ^1^H chemical shifts are in accordance with the known deprotonation of the central amine group of the complexed diethylenetriamine ligand. In ^13^C NMR spectra, two separate sets of resonances are observed for the chloro and the hydroxo complex of gold(III) diethylenetriamine. The hydroxo complex [Au(dien-H)OH^+^ shows exchange effects in ^13^C NMR spectra. Variable temperature studies show the carbon atoms next to the central secondary amine to be inequivalent and each present in two different environments that are in intermediate to fast exchange on the NMR time-scale.


### [AU(DIEN)Cl]Cl$_2$: EXCHANGE PHENOMENA OBSERVED BY $^1$H AND $^{13}$C NMR SPECTROSCOPY

Sabine L. Best*[1], Zijian Guo, Milos I. Djuran and Peter J. Sadler[2]

[1] Department of Chemistry, Birkbeck College, University of London, 29 Gordon Square, London WC1H OPP, UK
[2] Department of Chemistry, University of Edinburgh, West Mains Road, Edinburgh EH9 3JJ, UK

The solution behaviour of the square-planar gold(III) complex [Au(dien)Cl]Cl$_2$ (dien = 1,5-diamino-3-azapentane) was investigated by $^1$H and $^{13}$C NMR spectroscopy. We have found that $^1$H NMR spectra of [Au(dien)Cl]Cl$_2$ are characterised by exchange behaviour over the whole pH range, and some exchange effects are also seen in $^{13}$C NMR spectra of the deprotonated hydroxo derivative of the complex in alkaline solution. An exchange rate of > 378 $s^{-1}$ was determined from $^1$H NMR spectra at pH* 2 (coalescence temperature 40 °C). In slightly acidic solutions of the complex, $^1$H chemical shifts are in accordance with the known deprotonation of the central amine group of the complexed diethylenetriamine ligand. In $^{13}$C NMR spectra, two separate sets of resonances are observed for the chloro and the hydroxo complex of gold(III) diethylenetriamine. The hydroxo complex [Au(dien-H)OH]$^+$ shows exchange effects in $^{13}$C NMR spectra. Variable temperature studies show the carbon atoms next to the central secondary amine to be inequivalent and each present in two different environments that are in intermediate to fast exchange on the NMR time-scale.

### INTRODUCTION

Gold(III) has recently received much attention because of its possible involvement in the biological action/side-effects of anti-arthritic gold(I) drugs[0,1]. It has been shown that gold(III) is a reactive metabolite of gold(I) in mice[2] and humans[3]. The oxidation of gold(I) to gold(III) has been demonstrated *in vitro* and *in vivo*[4,5,6].

The known gold (III) chelate complex [Au(dien)Cl]Cl$_2$ is of special interest because of its potential as a probe for gold(III) binding sites on biological molecules, *e.g.* nucleotides[7] and peptides[8].

Chlorodiethylenetriaminegold(III) chloride was first prepared in 1963[9] and the crystal structure was solved in 1986[10]. In the solid state, the complex is pseudooctahedral with the nitrogens of the diethylenetriamine ligand occupying three of the equatorial coordination sites around gold(III) and one chlorine atom occupying the fourth coordination site in the plane. Two additional chlorine atoms are situated in axial positions with considerably longer Au-Cl bond lengths[11].

In aqueous solution, the complex [Au(dien)]$^{3+}$ has been shown to take part in both acid-base and hydrolytic equilibria[10]. The complex loses a proton from one of the nitrogen ligands with a p$K_a$ value[10] of 4.0 in 0.5 M $ClO_4^-$ or 4.7 in 0.5 M $Cl^-$ according to equation 1.

$$[Au(dien)Cl]^{2+} \leftrightarrow [Au(dien\text{-}H)Cl]^+ + H^+ \qquad (1)$$

It has been confirmed that deprotonation occurs from the central, secondary nitrogen in a single-crystal X-ray diffraction study in which both the crystal structures of [Au(dien)Cl]Cl[ClO$_4$] and [Au(dien-H)Cl][ClO$_4$] were solved[11].

In neutral to basic solution, the chloride at the fourth coordination site is replaced by hydroxide[10] (equation 2).

$$[Au(dien\text{-}H)Cl]^+ + H_2O \leftrightarrow [Au(dien\text{-}H)(OH)]^+ + H^+ + Cl^- \qquad (2)$$

We have used [Au(dien)Cl]Cl$_2$ in NMR studies of the interaction of gold(III) with peptides[9]. The complex has not yet been investigated in detail by NMR spectroscopy and therefore we have studied both $^1$H NMR and $^{13}$C NMR spectra of aqueous solutions of the complex.

### MATERIALS AND METHODS

### Preparation of [Au(dien)Cl]Cl$_2$

The complex was prepared by the method of Baddley et al.[10] (Found: C, 12.04; H, 3.03; N, 10.23. Calc. for $C_4H_{13}AuCl_3N_3$: C, 11.82; H, 3.22; N, 10.34%).

### NMR Spectroscopy

For $^{13}C$ NMR studies, a 50 mM solution of $[Au(dien)Cl]Cl_2$ in $D_2O$ was prepared with sodium 3-(trimethylsilyl)tetradeuteriopropionate (TSP-$d_4$) as internal reference. The pH of the solution was adjusted using NaOD and $DNO_3$ solutions. Measurements of pH were made using an Aldrich micro combination electrode and Corning 240 meter, calibrated with Aldrich buffer solutions. Readings of the pH meter for $D_2O$ solutions were not corrected for deuterium isotope effects and are designated as pH* values. Broad-band proton decoupled $^{13}C$-{$^1H$} NMR spectra were accumulated overnight at 67.8 MHz on a JEOL GSX270 spectrometer and typically processed using an exponential function equivalent to a line-broadening of 5 Hz. The solution was kept frozen between experiments.

For $^1H$ NMR studies, 10 mM solutions of $[Au(dien)Cl]Cl_2$ in $D_2O$ with dioxane as internal reference ($\delta$ 3.768 relative to TSP-$d_4$ at 295 K) were used and spectra accumulated at 270 MHz. Spectra were typically processed using an exponential function equivalent to a line-broadening of 0.4 Hz. Typical pulsing conditions were. spectral width 3.2 KHz, pulse width 45°, relaxation delay 2.2 s, 16 K datapoints, 128 transients. The pH titration curves were fitted to a modified form of the Hill equation[12] using the program KALEIDAGRAPH[13] on an Apple Macintosh computer.

A 100 mM solution of $[Au(dien)Cl]Cl_2$ in 0.6 ml $D_2O$ was used for the 2D [$^1H$,$^{13}C$] HSQC NMR experiment. The pH of the solution was adjusted to 1.5 using 1 M DCl. The 2D [$^1H$,$^{13}C$] HSQC NMR spectrum was recorded at 298 K using the sequence of Stonehouse et al.[14] on a Varian INOVA 600 spectrometer ($^1H$ 600 MHz, $^{13}C$ 150.8 MHz). The $^{13}C$-spins were decoupled by irradiation with the GARP-1 sequence (optimized for $^1J$(C, H) = 140 Hz) during acquisition. Water suppression was achieved by pulsed-field gradients. Typically, 128 scans were acquired for each of 64 increments of $t_1$ and the final resolution was 4 Hz/point for the F2 dimension and 8 Hz/point for the F1 dimension.

### RESULTS

### $^{13}C$ NMR Studies

Figure 1 shows the numbering system used for the carbon atoms in the complex $[Au(dien)Cl]^{2+}$.

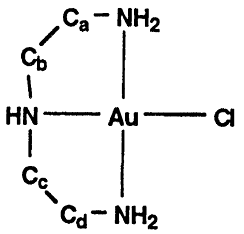

Figure 1: Numbering system for the carbon atoms in $[Au(dien)Cl]^{2+}$

Proton-decoupled $^{13}C$ NMR spectra of $[Au(dien)Cl]Cl_2$ at selected pH* values are shown in Figure 2 and a plot of chemical shift values of the various $^{13}C$ resonances versus pH* is shown in Figure 3. Up to pH* 5.2, only two $^{13}C$ signals were observed indicating that carbon atoms $C_a$ and $C_d$ plus $C_b$ and $C_c$ are equivalent. The more downfield of the two resonances (peak 1) was assigned to the equivalent carbon atoms $C_b$ and $C_c$ next to the secondary amine. Peak 2 was assigned to the equivalent carbon atoms $C_a$ and $C_d$ next to the primary amine groups. This was based on the previous assignment of resonances for the palladium(II)-diethylenetriamine complex on the basis of chemical shift data[15] for $CH_3NHCH_2CH_2NHCH_3$ and $NH_2CH_2CH_2NH_2$ and is in agreement with some previously reported carbon-13 data for the complex[16]. Both the resonances were pH dependent with peak 1 showing a downfield shift of 4.9 ppm and peak 2 an upfield shift of 1.5 ppm upon pH increase from pH* 3.0 to pH* 6.5 (Figures 2 and 3). The shifts were fitted to a $pK_a$ value of 4.74 ± 0.04 and attributed to the deprotonation of the central secondary amine group as suggested by Baddley et al.[10] and confirmed by X-ray crystallographic studies[12].

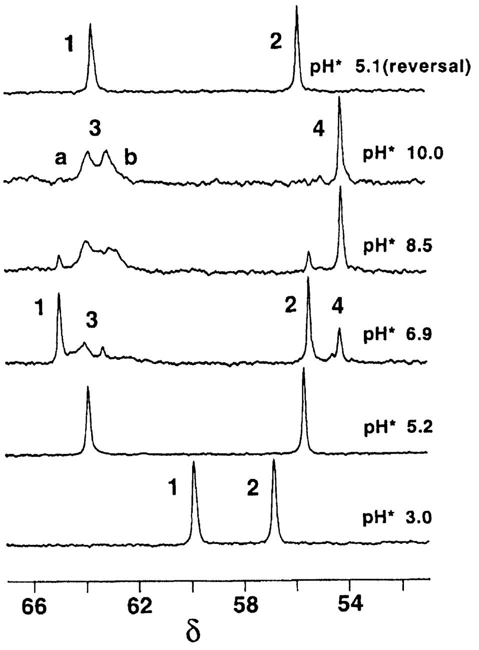

Figure 2: 67.8 MHz $^{13}C$-{$^{1}H$} NMR spectra of 50 mM $[Au(dien)Cl]Cl_2$ in $D_2O$ at various pH* values, the spectrum of a sample after reversal from pH* 10 to pH* 5.1 is also shown

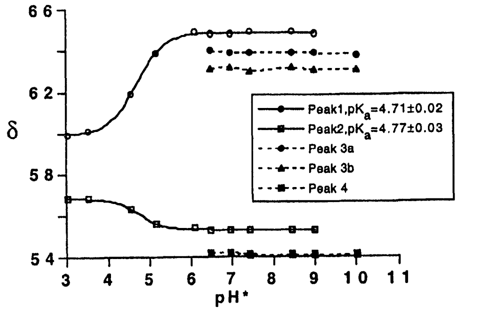

Figure 3: $^{13}C$ chemical shift values versus pH* for $[Au(dien)Cl]Cl_2$ in $D_2O$; the curves for peaks 1 and 2 are computer best-fits

In spectra recorded at pH* 6.1 and higher, new resonances (two broad peaks 3a, 3b and one sharp peak 4) appeared and increased in intensity with increasing pH until they were the only resonances observed at pH* 10.0. From previous studies[10], the complex would be expected to be fully hydrolysed by pH 10, so that the new peaks appearing at pH* ≥ 6.1 were assigned to the deprotonated hydroxo derivative $[Au(dien\text{-}H)OH]^+$. From their chemical shift values, the downfield (broad) resonances (peaks 3a and 3b) were again assigned to the carbon atoms $C_b$ and $C_c$ next to the secondary, central amine, while the upfield, sharp peak (peak 4) was assigned to the carbon atoms $C_a$ and $C_d$ next to the primary amines. The changes in the spectra of $[Au(dien)Cl]^{2+}$ were reversible as lowering the pH from pH* 10.0 to pH* 5.1 yielded the same spectrum as before the pH increase.

Increasing the temperature of a solution of $[Au(dien)Cl]Cl_2$ in $D_2O$, pH* 9.9 from 22 °C to 60 °C led to a clear sharpening of peaks 3a and 3b in $^{13}C$-{$^{1}H$} NMR spectra (Figure 4). Temperatures higher than 60 °C could not be employed due to the instability of the complex.

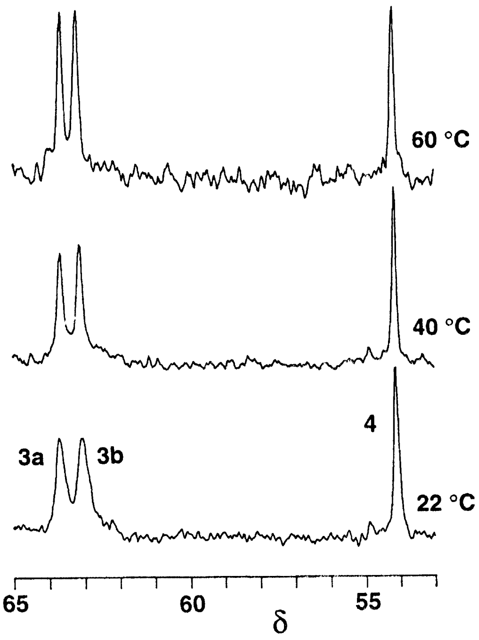

Figure 4: 67.8 MHz $^{13}C$-{$^{1}H$} NMR spectra of 50 mM [Au(dien-H)(OH)]$^+$ in $D_2O$, pH* 9.9, at various temperatures

### $^1$H NMR Studies

$^1$H NMR spectra of [Au(dien)Cl]$Cl_2$ in $D_2O$ were characterized by exchange processes over the whole pH range (Figure 5). In the pH* range 3.0 to 5.9, only two broad peaks were seen which showed pH-dependent shifts. Peak 1 ($\Delta\delta$ = 1.09 ppm in the pH* range 3.0 to 7.0) was broad over the whole investigated pH range. Peak 2 ($\Delta\delta$ = 0.27 ppm in the pH* range 3.0 to 7.0) was broad at low pH values but turned into a relatively sharp triplet in spectra of pH* 4 and above. The pH-dependent shifts of those peaks were again attributed to the deprotonation of the central secondary amine group. Peak 1 was assigned to the methylene protons of $C_b$ and $C_c$ because of its large pH-dependent shift upon deprotonation of the central amine; peak 2 with a smaller shift difference was assigned to the protons attached to $C_a$ and $C_d$.

An increase of the temperature from 22 °C to 60 °C of a solution of [Au(dien)Cl]$Cl_2$ in $D_2O$ at pH* 3.2 resulted in the appearance of two triplets in place of the very broad resonances seen at this pH* value at 22 °C (Figure 6). Upon lowering the pH* to 2.0 at 22 °C, both peak 1 and peak 2 split into two broad resonances (peaks 1a, 1b and 2a, 2b respectively, Figure 7). Upon temperature increase, those peaks broadened and coalesced (Figure 7) with coalescence temperatures of ca. 30 °C for peak 2 and ca. 40 °C for peak 1. The calculated exchange rates at these temperatures from the chemical shift difference at 22 °C were > 118 $s^{-1}$ for peak 2 and > 378 $s^{-1}$ for peak 1.

At pH* values of 7.0 and above, additional $^1$H NMR resonances were observed. Contrary to the $^{13}$C spectra, where a complete set of new peaks appeared for the hydroxo species at higher pH values, the difference between chloro and hydroxo species was not as clear in $^1$H NMR spectra. Peak 1 was observable as a broad resonance up to pH* 12.1 and must therefore represent protons in the chloro- as well as the hydroxo species. Two very broad resonances (peaks 3a and 3b) appeared on either side of peak 1 in spectra between pH* 7.0 and 10.9 (Figure 5). New resonances also appeared around peak 2 at pH* values of 7.9 and higher (peak 4). They were not of equal intensity and were difficult to follow.

Variable temperature experiments of a solution at pH* 9.9 (Figure 8) showed peak 1 sharpening to form a still broad triplet at higher temperatures, while the very broad peaks 3a and 3b broadened and coalesced at *ca.* 40 °C. The exchange rate at this temperature was > 443 $s^{-1}$ from the chemical shift difference at 22 °C. At the same pH* value, the resonances for the methylene protons of $C_a$ and $C_d$ (peaks 2 and 4) broadened upon temperature increase.

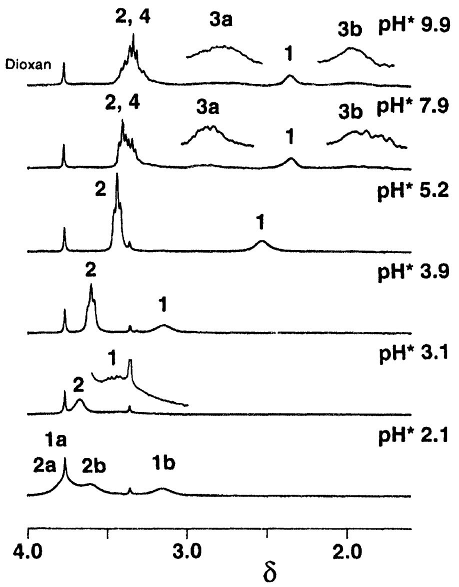

Figure 5: 270 MHz $^1$H NMR spectra of 10 mM [Au(dien)Cl]$Cl_2$ in $D_2O$ at various pH* values

### [$^1$H,$^{13}$C] NMR Spectroscopy

The 2D HSQC NMR spectrum of [Au(dien)Cl]$Cl_2$ at pH* 1.5 showed four cross-peaks at 3.90/58.30, 3.64/58.30, 3.89/61.05 and 3.20/61.05 ppm (Figure 9). The former two cross-peaks can be assigned to the two -$CH_2$-$NH_2$ groups ($C_a$ and $C_d$) and the latter two to the -$NHCH_2$ ($C_b$ and $C_c$) groups of the dien ligand. Only two sets of carbon resonances were observed for the dien ligand at pH* 1.5. It is notable that the two protons of $NHCH_2$ had a $^1$H chemical shift difference of *ca.* 0.7 ppm.

### DISCUSSION

The $pK_a$ value of 4.74 ± 0.04 (in $D_2O$, 22 °C, I -> 0) determined in the present study for the deprotonation of the central, secondary amine group is in a similar range to the one determined by Baddley et al.[10] ($pK_a$ = 4.0 in $H_2O$, 25 °C, I = 0.5 with $ClO_4^-$). The difference between the two values is likely to be due to the different conditions, *i.e.* a combination of different ionic strength and deuterium isotope effect. The largest $^{13}$C shifts are seen on the carbon atoms $C_b$ and $C_c$ next to the deprotonating group. This was also found in the complex [Pt(dien)($Me_2SO$)]$^{2+}$, in which the central amine group deprotonates with a $pK_a$ value[17] of 11.94. The two-dimensional [$^1$H,$^{13}$C] HSQC NMR experiment confirms the assignment of the respective $^1$H and $^{13}$C resonances in the one-dimensional spectra.

Exchange processes in the hydroxo species [Au(dien-H)(OH)]$^+$ account for the broad appearance of peaks 3a and 3b in the $^{13}$C spectra (Figure 2). Upon increase in temperature from 22 °C to 60 °C (Figure 4), the two broad peaks become sharper. The sharpening of the resonances suggests that the two resonances are not exchanging with each other, otherwise signals would be expected to become broader at higher temperatures and eventually coalesce to a single peak. In contrast to this, an increasing sharpening of the peaks is observed that can only be explained by assuming that each of the two broad peaks is already an average of two resonances in intermediate to fast exchange on the NMR time-scale. The carbon atoms next to the terminal amines, $C_a$ and $C_d$ stay equivalent under the conditions used. This behaviour suggests that the carbon atoms $C_b$ and $C_c$ next to the central amine in [Au(dien-H)]$^{2+}$ (peaks 3a and 3b) are inequivalent in the hydroxo species and that each of the inequivalent carbon atoms $C_b$ and $C_c$ is present in two different environments that are in intermediate to fast exchange on the NMR time-scale. The different environments may be due to different conformations of the five-membered chelate rings which exist in two enantiomeric forms (λ and δ) with the interconversion between these structures proceeding via an envelope conformation[18].

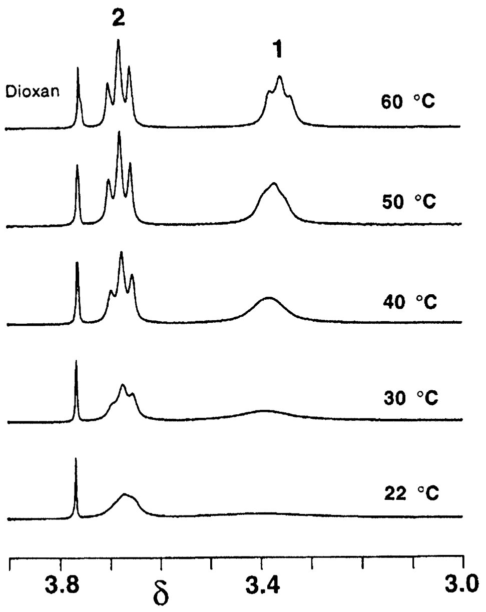

Figure 6: 270 MHz $^1$H NMR spectra of 10 mM $[Au(dien)Cl]Cl_2$ in $D_2O$, pH* 3.2, at various temperatures

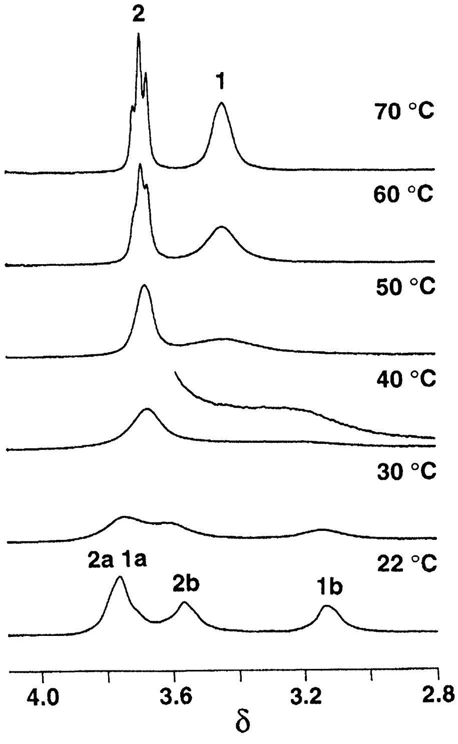

Figure 7: 270 MHz $^1$H NMR spectra of 10 mM $[Au(dien)Cl]Cl_2$ in $D_2O$, pH* 2.0, at various temperatures

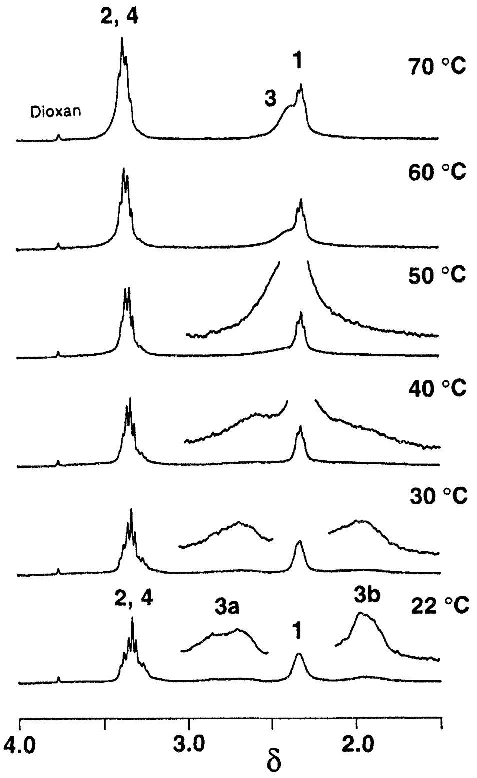

Figure 8: 270 MHz $^{1}H$ NMR spectra of 10 mM $[Au(dien)Cl]Cl_2$ in $D_2O$, pH* 9.9, at various temperatures

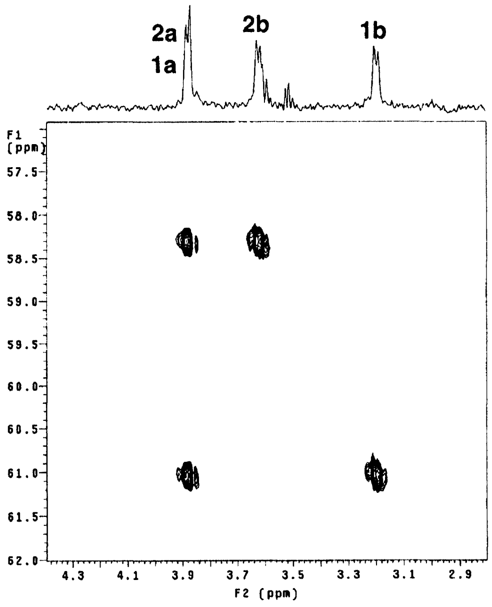

Figure 9: 2D $[^{1}H,^{13}C]$ HSQC NMR spectrum of 100 mM $[Au(dien)Cl]Cl_2$ in $D_2O$, pH* 1.5

However, the interconversion is usually believed to be too rapid to be observed[19]. Various explanations are possible for the inequivalence of $C_b$ and $C_c$ in the hydroxo-species. It might be due to the presence of a hydroxo-bridged dimer. The crystal structure of dimethylgold(III) hydroxide, a hydroxo-bridged tetrameric gold(III) complex, has been reported[20] and hydroxo-bridged complexes are well known for palladium(II) and platinum(II) complexes. Restricted rotation in a possible dimer could account for the inequivalence of the carbon atoms. Another possibility is hydrogen-bonding with axial hydroxide substituents around gold(III) which could render the molecule less symmetrical. It must be noted that the carbon atoms are equivalent in the chloro species $[Au(dien\text{-}H)Cl]^+$. In this complex, the axial substituents are chloride ions and they are thought to be replaced by hydroxide upon increasing the pH of the solution[10].

Proton NMR spectra of $[Au(dien)Cl]Cl_2$ show exchange-broadening over the whole pH range, *i.e.* both chloro and hydroxo complexes are affected. The broadening at low and at higher pH is likely to be caused by different mechanisms. At low pH (pH* 2.0, Figure 7), broadening could be explained most easily by a ring-opening and -closing equilibrium involving protonation of one of the terminal $NH_2$ groups. An equilibrium between unprotonated ring-closed and protonated ring-opened species of $[Au(dien)Cl]^{2+}$ has been suggested to occur in acidic solution on the basis of kinetic studies[21]. Ring opening at one end of the molecule in $[Au(dien)Cl]Cl_2$ would render the methylene groups of the two five-membered chelate rings inequivalent and broadening would occur if the exchange between ring-opened and ring-closed species was at an intermediate rate on the NMR time-scale. As the $H^+$ concentration decreases, ring-opening would be suppressed, consistent with the sharpening of the resonances for the methylene protons of $C_a$ and $C_d$ at pH* > 3 (peak 2 in Figure 5). However, the observation of only two carbon resonances at pH* 1.5 suggests that no ring-opening reaction occurs at that pH* value. The inequivalence of the $^1H$ resonances in strongly acidic solution must therefore be due to a different mechanism, possibly again an interconversion of different conformations of the five-membered chelate rings.

The exchange rates calculated from $^1H$ NMR temperature studies at pH* 2 and 10 are estimates because of the broadness of the resonances at the lowest temperatures studied. The maximum chemical shift difference of the exchanging protons in the two environments might be larger and the exchange rates are therefore lower limits.

Additional resonances in $^1H$ NMR spectra of $[Au(dien)Cl]Cl_2$ (peaks 3a, 3b and 4, Figure 5) occur above pH* 7 in solutions of the complex alone, but not in spectra of a mixture of $[Au(III)(Gly\text{-}Gly\text{-}L\text{-}His\text{-}H_{-2})]^+$ and the complex, in which the major species between pH* 6 and 12 is the bridged imidazole complex[9]. Therefore, those additional resonances are a characteristic of the hydroxo complex, possibly due to a hydroxo-bridged complex, rather than a feature of the diethylenetriamine ligand.

We have shown in this work that the seemingly simple complex of gold(III) with diethylenetriamine exhibits an extremely complex solution behaviour. More work is certainly needed to unravel the basis of the various exchange processes observed in $[Au(dien)]^{3+}$.

### ACKNOWLEDGEMENTS

This work was supported by the Wellcome Trust (Toxicology Studentship to S. L. B., Ref. No. 035931/Z/92/Z) and the Royal Society. We thank the University of London Intercollegiate Research Service (ULIRS) and the Medical Research Council Biomedical Centre for the provision of NMR facilities.

### REFERENCES

0. Best, S.L.; Sadler P.J. *Gold Bull.* **29**, 87-94 (1996).
1. Griem, P.; Gleichmann, E. *Curr. Opin. Immunol.* **7**, 831-838 (1995).
2. Schuhmann, D., Kubicka-Muranyi, M., Mirtschewa, J., Günther, J., Kind, P., Gleichmann, E. *J. Immunol.* **145**, 2132-2139 (1990).
3. Verwilghen, J.; Kingsley, G.H.; Gambling, L.; Panayi, G.S. *Arthritis Rheum.* **35**, 1413-1418 (1992).
4. Shaw, C.F. III, Schraa, S., Gleichmann, E., Grover, Y.P., Dunemann, L., Jagarlamudi, A. *Metal Based Drugs* **1**, 351-362 (1994).
5. Takahashi, K., Griem, P., Goebel, C., Gonzales, J., Gleichmann, E. *Metal Based Drugs* **1**, 483-496 (1994).

6. Goebel, C. Kubicka-Muranyi, M., Tonn, T., Gonzales, J., Gleichmann, E. *Arch. Toxicol.* **69**, 450-459 (1995).
7. Feldmann, G.; Lippert, B. *Abstracts EUROBIC II*, 122 (1994).
8. Best, S.L.; Djuran, M.I.; Sadler, P.J. *in preparation.*
9. Baddley, W.H.; Basolo, F.; Gray, H.B.; Nölting, C.; Poë, A.J. *Inorg. Chem.* **2**, 921-928 (1963).
10. Elder, R.C.; Watkins, J.W. *Inorg. Chem.* **25**, 223-226 (1986).
11. Nardin, G.; Randaccio, L.; Annibale, G.; Natile, G.; Pitteri, B. *J. Chem. Soc. Dalton Trans.* 220-223 (1980).
12. Markley, J.L. *Acc. Chem. Res.* **8**, 70-80 (1975).
13. KALEIDAGRAPH, Synergy Software, Reading, PA, (1994).
14. Stonehouse, J., Shaw, G.L., Keeler, J., Laue, E.D. *J. Magn. Res. Ser A* **107**, 178-184 (1994).
15. Chin, P.-K.F.; Hartley, F.R. *Inorg. Chem.* **15**, 982-984 (1976).
16. Gladkaya, A.Sh.; Don, G.M.; Evstaf'eva, O.N.; Golovaneva, I.F. *Russ. J. Inorg. Chem.* **36**, 683-687 (1991).
17. Romeo, R.; Minniti, D.; Alibrandi, G.; De Cola, L.; Tobe, M.L. *Inorg. Chem.* **25**, 1944-1947 (1986).
18. Hawkins, C.J.; Palmer, J.A. *Coord. Chem. Rev.* **44**, 1-60 (1982).
19. Hawkins, C.J., *Absolute Configuration of Metal Complexes*, Wiley-Interscience, New York, pp 105-107 (1971).
20. Glass, G.E., Konnert, J.H., Miles, M.G., Britton, D., Tobias, R.S. *J. Am. Chem. Soc.* **90**, 1131-1138 (1968).
21. Annibale, G.; Natile, G.; Cattalini, L. *J. Chem. Soc. Dalton Trans.* 1547-1552 (1976).

**Received: September 15, 1998 -**
**Accepted in final form: October 9, 1998**

